# Sex- and Age-Dependent Associations between *Parabacteroides* and Obesity: Evidence from Two Population Cohort

**DOI:** 10.3390/microorganisms11082087

**Published:** 2023-08-15

**Authors:** Feng Zhang, Xiru Zhang, Jingxiang Fu, Zhuo Duan, Wen Qiu, Yijia Cai, Wenjun Ma, Hongwei Zhou, Yuming Chen, Jusheng Zheng, Yan He

**Affiliations:** 1 Microbiome Medicine Centre, Department of Laboratory Medicine, Zhujiang Hospital, Southern Medical University, Guangzhou 510515, China; zhangfch0523@163.com (F.Z.);; 2 Guangdong Provincial Institute of Public Health, Guangdong Provincial Centre for Disease Control and Prevention, Guangzhou 510440, China; 3 Guangdong Provincial Key Laboratory of Food, Nutrition and Health, Department of Epidemiology, School of Public Health, Sun Yat-sen University, Guangzhou 510275, China; 4 Key Laboratory of Growth Regulation and Translational Research of Zhejiang Province, School of Life Sciences, Westlake University, Hangzhou 310024, China; zhengjusheng@westlake.edu.cn; 5 State Key Laboratory of Organ Failure Research, Southern Medical University, Guangzhou 510515, China; 6 Guangdong Provincial Clinical Research Center for Laboratory Medicine, Guangzhou 510033, China

**Keywords:** *Parabacteroides*, obesity, weight change, sex- and age-dependent, longitudinal cohort study

## Abstract

*Parabacteroides* levels are reported to be low in obese individuals, and this genus has shown an anti-obesity capacity in animal studies. Nevertheless, the relationship between *Parabacteroides* and obesity in different subpopulations, e.g., with respect to age and sex, and its association with subsequent weight change have rarely been explored. The cross-sectional associations of *Parabacteroides* genus- and species-level *OTU* abundance with obesity were explored in the Guangdong Gut Microbiome Project (GGMP), which included 5843 adults, and replicated in the Guangzhou Nutrition and Health Study (GNSH), which included 1637 individuals. Furthermore, we assessed the prospective associations of *Parabacteroides* and its main *OTUs’* abundance with the subsequent changes in body mass index (BMI) in the GNSH. We found that *Parabacteroides* was inversely associated with obesity among females and participants aged 40–69 years in the GGMP and the replicated cohort in the GNSH. After a 3-year follow-up, there was no significant correlation between *Parabacteroides* and the subsequent changes in BMI. However, *Seq4172* (*P. johnsonii*) showed a negative correlation with subsequent BMI changes in the female and middle-aged (40–69 years) subpopulations. Overall, our results indicate that *Parabacteroides* have an inverse relationship with obesity and that *Seq4172* (*P. johnsonii*) have a negative association with subsequent changes in BMI among females and middle-aged populations in perspective analyses.

## 1. Introduction

Obesity has become one of the greatest public health problems globally. The prevalence of obesity among adults aged 20 years and older reached 39.8% according to the latest available year of the WHO reports [1]. Several previous studies have demonstrated that obesity or overweight are associated with an increased risk of new-onset chronic noncommunicable diseases (NCDs), such as cardiovascular diseases, diabetes, cancer, and hypertension, causing approximately 2.8 million deaths in 2021 [2,3,4]. Additionally, obesity affects the course and effectiveness of infectious diseases [5]. Although living a healthy lifestyle (e.g., having a balanced diet, regularly engaging in exercise, and/or limiting alcohol intake), using weight-reducing medicine, and liposuction have been promoted for the prevention or treatment of obesity [6], the prevalence of obesity is still rising. It is estimated that 50% of the global population will be obese by the year 2050 [7], and this issue is particularly troubling in China, with an incredible obesity rate that is projected to increase from 39.8% in 2018 to 51.2% in 2035 [8]. Therefore, the identification of a novel intervention target is an emerging research topic in relation to combatting the obesity epidemic.

The role of gut microbiota dysbiosis in the pathogenesis of obesity and metabolic disorders has attracted extensive attention based on evidence from rodent models and human studies [9,10]. Among the complex gut microbiota, one bacterial genus, that is, *Parabacteroides*, has been scrutinized due to its potential anti-obesity activity. Wu T R et al. reported that *Parabacteroides* levels were reduced in HFD-fed mice and that an oral treatment of *Parabacteroides* could reduce obesity by increasing adipose tissue thermogenesis [11], while Wang K et al. found that *Parabacteroides* alleviated obesity by modulating host metabolism via increasing the production of the secondary bile acids UDCA and succinate, thereby enhancing the excretion of hepatic lipids, inhibiting hepatic long-chain FFA uptake, and restoring gut mucosal integrity in rodent models [12]. Additionally, De Wouters d’Oplinter A et al. revealed that *Parabacteroides* could alleviate obesity by limiting food intake via modulating the dopaminergic mesocorticolimbic system [13]. However, there were inconsistencies in the findings regarding the relationship between *Parabacteroides* and obesity in population-based studies. Using 16s rRNA sequencing analysis, Vanessa Palmas et al. observed that overweight/obese patients exhibited a significant decrease in *Parabacteroides* abundance compared with normal controls among 46 pairs of Italians [14]. Some studies have revealed that the abundance of *Parabacteroides* is positively correlated with unhealthier eating behavior and poorer weight loss maintenance among bad vs. good Roux-en-Y gastric bypass responder patients [15]. Furthermore, the enrichment of *Parabacteroides* has been reported to be associated with the development of early-life obesity among Hispanic children [16]. These controversies indicate the necessity of exploring the relationship between Parabacteroides and obesity in large-scale, population-level datasets, especially in prospective cohorts. Moreover, it would also be interesting to determine if the association between *Parabacteroides* and obesity exhibits age or sex dependency, which might partially explain the inconsistency between different studies and even guide precise applications in the future.

In this study, we first used the population-based cross-sectional dataset including nearly six thousand individuals from the Guangdong Gut Microbiome Project (GGMP) to assess how *Parabacteroides* was correlated with obesity, in addition to analyzing such correlations in different subpopulations (with respect to age and sex), and replicated such associations with respect to 1637 participants from the Guangzhou Nutrition and Health Study (GNHS). Subsequently, we evaluated the prospective associations between *Parabacteroides* and weight change in a longitudinal dataset from the GNHS.

## 2. Method

### 2.1. Study Design and Participants

**Figure 1:**
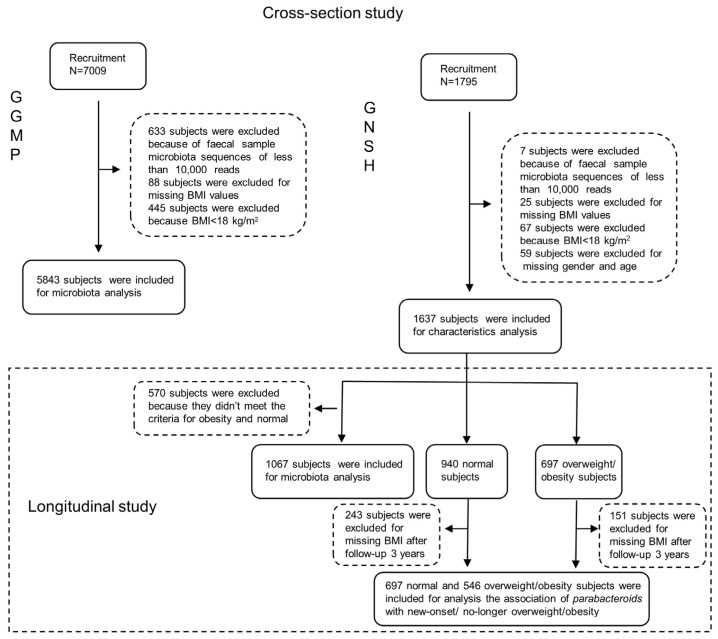
The flow chart of participant enrolment in GGMP and GNSH.

The Guangdong Gut Microbiome Project (GGMP) was conducted in 14 districts of Guangdong Province (southern China) between 2015 and 2017, in which 7009 participants aged 18–97 years old were randomly enrolled via the probability-proportional-to-size sampling strategy. The details of the GGMP have been described previously [17,18]. All participants signed informed consent, and the study was approved by the Ethical Review Committee of the Chinese Centre for Disease Control and Prevention (No. 201519-A). After the exclusion of individuals whose stool sample sequences provided fewer than 10,000 reads (*n* = 633), those with the missing values in terms of height or weight (*n* = 88), and those with a BMI less than 18.5 kg/m^2^ (*n* = 445), a total of 5843 participants were included in the analysis. The Guangzhou Nutrition and Health Study (GNHS) was a longitudinal study that was established in 2011, for which follow-ups were conducted approximately every 3 years. It was approved by the Ethics Committee of the School of Public Health at Sun Yat-sen University (NCT03179657). A total of 1795 stool samples and the origin values of BMI (BMI_T0) were collected as a baseline; a detailed description of this process was provided in previous research [19]. After the exclusion of participants with low-quality stool samples (*n* = 7), those with missing values in terms of height or weight (*n* = 25) or with a BMI less than 18.5 kg/m^2^ (*n* = 67), and those with missing data on characteristic information (*n* = 59), our final analysis included 1637 individuals (Figure 1). Subsequently, the initial BMI (BMI_T1) values among these individuals were obtained after 3 years of follow-ups.

### 2.2. The Assessment of Obesity/Overweight, BMI Change, and Other Covariates

Anthropometric data, including height, weight, waist circumference (WC), systolic blood pressure (SBP), and diastolic blood pressure (DBP), were measured by trained faculty staff. BMI was calculated by dividing a participant’s weight by the square of his or her height in meters (kg/m^2^). The participants, stemming from the GGMP and GNHS, were classified as normal weight (18.5 ≤ BMI < 24.0 kg/m^2^), overweight (24.0 ≤ BMI < 28.0 kg/m^2^), or obese (BMI ≥ 28.0 kg/m^2^) according to the criteria of the Working Group on Obesity in China (WGOC) [20]. The change in BMI (ΔBMI) was calculated by subtracting the BMI in the first follow-up by the initial BMI of the participants from the GNHS. Information on the sociodemographic features, lifestyles, diet, and medications of each patient were collected via face-to-face questionnaire interviews. Fasting venous blood samples were collected by registered nurses and stored in a −80 °C freezer prior to analysis. Fasting blood glucose (FBG), glycated hemoglobin (HbAlc), total cholesterol (TC), triglyceride (Tg), high-density lipoprotein cholesterol (HDL-C), low-density lipoprotein cholesterol (LDL-C), and uric acid (UA) levels were measured using a Hitachi 7600 (Hitachi, Tokyo, Japan) automatic biochemical analyzer at the Chinese Centre for Disease Control and Prevention.

### 2.3. Stool Samples and Bioinformatics Analysis

All procedures in both studies, including stool sample collection and transportation and DNA extraction, amplification, and sequencing, followed the previous experimental protocols [10,11,13]. Raw sequences were pre-processed and analysed using the pipeline developed by our team (https://github.com/SMUJYYXB/GGMP-Regional-variations, accessed on 21 September 2018) and Quantitative Insights Into Microbial Ecology software 2 (QIIME2) [21]. Then, we used Deblur to denoise and generate sub-operational taxonomic units (*OTUs*). PyNAST and FastTree were used to align the sequences and build a phylogenetic tree. We used the RDP classifier in QIIME with the GreenGenes (version 13.8) databases for taxonomic assignment. For unknown species, we further aligned their representative reads to an rRNA/ITS database using BLAST (https://blast.ncbi.nlm.nih.gov/Blast.cgi, accessed on 22 June 2023) in order to assign species based on the best match. Samples with fewer than 10,000 sequences were discarded in this analysis, and the remaining samples were rarefied to 10,000 reads. In the GNHS, the 16S rRNA V3-4 region was sequenced, and to ensure that the analysis pipeline of the two cohorts was consistent, we extracted the V4 region of 16S rRNA from V3-4 reads and then used the same pipeline for downstream analysis.

### 2.4. Statistical Analysis

*Parabacteroides* and its main *OTUs*’ abundance were classified as quartiles. The basic characteristics of the eligible participants were summarized, stratified according to *Parabacteroides* abundance quartiles, as numbers (percentage [%]) for categorical variables, means (standard deviation [SD]) for normally distributed variables, and medians (interquartile ranges) for skewed variables. The correlation between *Parabacteroides* along with its main *OTUs*’ abundance and the characteristics potentially related to obesity/overweight, including sociodemographic information (age, and sex), anthropometric measurements (BMI, WC, SBP, and SDP), biochemical indicators (FBG, HbA1c, TC, Tg, HDL-C, LDL-C, and UA), lifestyle habits (fruit or vegetable intake, grain intake, livestock meat intake, alcohol (high-alcohol liquor, low-alcohol liquor, wine, yellow rice wine, rice wine, and beer) intake, carbonated beverage or fruit drink consumption, smoking status, sedentary time, and sleeping duration), and Bristol stool type were examined using the Spearman correlation coefficients among the participants from the GGMP. The Benjamini–Hochberg method was used to adjust the *p* values. Logistic regression models were developed to estimate the odds ratios (ORs) and 95% confidence intervals (95% CIs) for obesity according to the quartiles of the abundance of *Parabacteroides* and its main *OTUs*, using the lowest quartile (Q1) as the reference group, among the participants from the GGMP and GNHS, respectively. For the above analyses, we tested two models: model 1, which was unadjusted, and model 2, which was adjusted for age and sex. We conducted a stratified analysis to assess the potential modification effects of sex (male and female) and age (18–39-, 40–69-, and 70–90-years old). Furthermore, we longitudinally explored whether the *Parabacteroides* along with its *OTUs*’ abundance quartiles were associated with new-onset overweight/obesity, or the cessation overweight/obesity, using the Logistic regression models with ORs and 95% CIs concerning a sample of 697 normal participants and a sample of 546 overweight/obese participants at the initial stage of the GNHS after the exclusion of participants with missing values with respect to weight or height at the first follow-up. Additionally, we explored whether *Parabacteroides* and its main *OTUs*’ abundance were associated with changes in BMI (ΔBMI) using a linear regression model. Similarly, we developed two models—namely, model 1, which was unadjusted, and model 2, which was adjusted for age and sex—and performed a stratified analysis to assess the potential modification effects exerted by sex (male and female) and age (40–69- and 70–90-years-old). All statistical analysis and data plotting were performed using the statistical software product R (version 4.1.1). *p* values less than 0.05 (two-sided) were considered statistically significant.

## 3. Results

### 3.1. Parabacteroides Distribution and Covariates in the Studied Population

Table 1 presents the characteristics of the participants from the GGMP stratified according to *Parabacteroides* abundance quartiles. Of the 5843 individuals (mean (SD) age, 52.98 (14.34) years), 2598 of the participants (44.5%) were male. Compared with participants with lower *Parabacteroides* abundance, those with higher *Parabacteroides* abundance were more likely to have lower BMI, WC, SBP, DBP, FBG, TC, Tg, and UA levels. (Table 1). Among the 1637 participants (mean (SD) age, 63.98 (6.07) years) from the GNHS, 34.0% were male. There were no significant differences in the basic characteristics stratified according to *Parabacteroides* abundance quartiles, except for the Tg levels (Supplementary File S1: Table S1).

**Figure 2:**
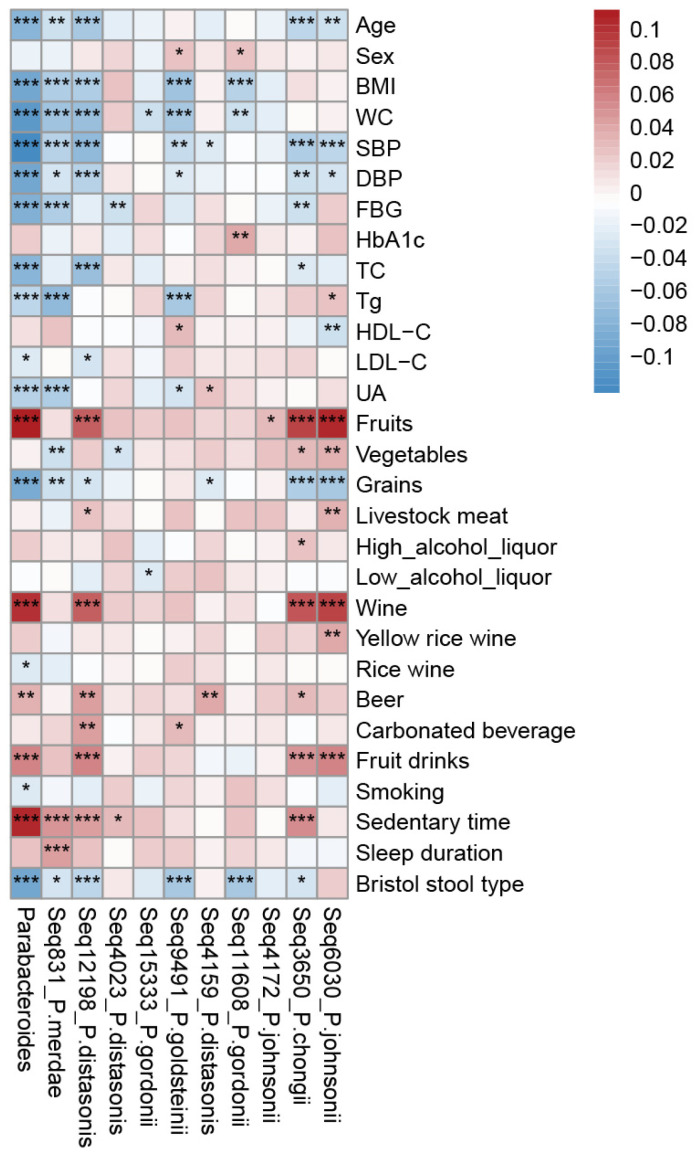
Host parameters related to *Parabacteroides*. Spearman correlations between *Parabacteroides* and its main OTUs. Color depth indicates the Spearman correlation coefficients. Red indicates a positive correlation, while blue indicates a negative correlation. * FDR < 0.05, ** FDR < 0.01, and *** FDR < 0.001. Abbreviations: BMI, body mass index; WC, waist circumference; SBP, systolic blood pressure; DBP, diastolic blood pressure; FBG, fasting blood glucose; TC, total cholesterol; HDL-C, HDL cholesterol; LDL-C, LDL cholesterol; UA, uric acid; BUN, blood urea nitrogen.

The detection rate of *Parabacteroides* was 97.50% (5697/5843) in the GGMP (Additional File S1: Figure S1A). The mean relative abundance of *Parabacteroides* was 0.96%, ranking 24th at the genus level. After denoising, there were ten sequence-level *Parabacteroides* units detected, namely, *Seq831* (*P. merdae*), *Seq12198* (*P. distasonis*), *Seq4023* (*P. distasonis*), *Seq15333* (*P. gordonii*), *Seq9491* (*P. goldsteinii*), *Seq4159* (*P. distasonis*), *Seq11608* (*P. gordonii*), *Seq4172* (*P. johnsonii*), *Seq3650*(*P. chongii*), *and Seq6030*(*P. johnsonii*), which accounted for more than 80% of the abundance of *Parabacteroides* (Supplementary File S1: Figure S1B). *Seq831* (*P. merdae*) was the most prevalent (4676/5843) and the most abundant (mean relative abundance: 0.41%) at the sequence level (Supplementary File S1: Figure S1B). The abundance of *Parabacteroides* decreased with an increasing age, BMI, WC, SBP, SDP, FBG, TC, Tg, LDL-C, UA, grain intake, and Bristol stool type but increased with increasing fruits intake; wine, beer, and fruit drink consumption; and sedentary time (Figure 2). At the sequence-level, *Seq831* (*P. merdae*), *Seq12198* (*P. distasonis*), and *Seq9491* (*P. goldsteinii*) were negatively correlated with blood pressure, BMI, WC, and Bristol stool type, whereas *Seq4023* (*P. distasonis*) was positively correlated with BMI and WC (Figure 2).

In GNSH, *Parabacteroides* was detected in 94.56% of all samples (Supplementary File S1: Figure S2A). After denoising, there were six sequence-level *Parabacteroides* units, among which *Seq831* (*P. merdae*), *Seq12198* (*P. distasonis*), *Seq4023* (*P. distasonis*), *Seq6030* (*P. johnsonii*), *Seq15333*(*P. gordonii*), *and Seq4172* (*P. johnsonii*) accounted for more than 80% of the abundance of *Parabacteroides* (Supplementary File S1: Figure S2B). Moreover, *Seq831* (*P. merdae*) was dominant in both the GGMP and GNSH cohorts.

### 3.2. The Association between Parabacteroides Abundance and Obesity

**Figure 3:**
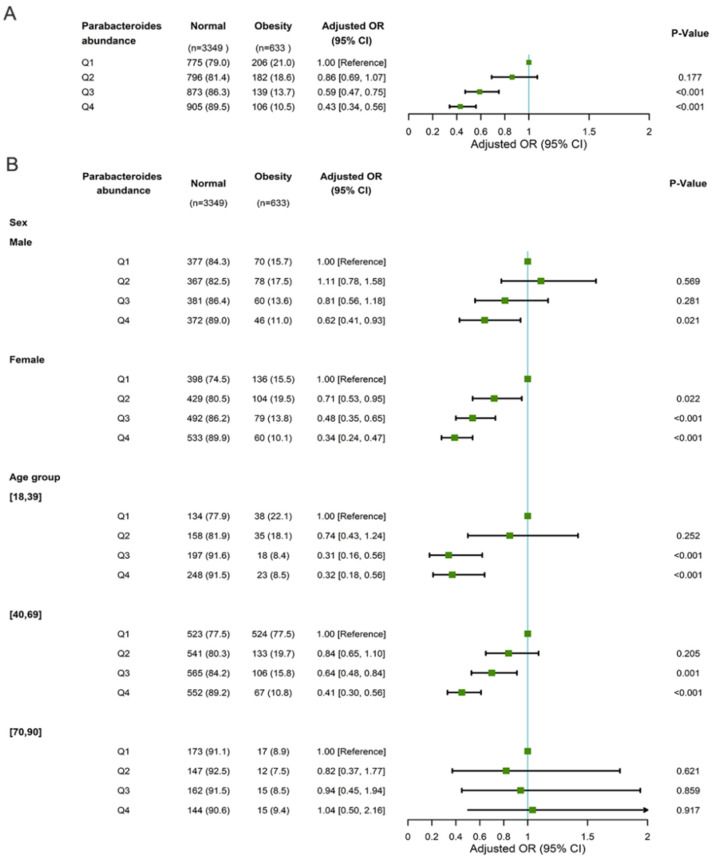
Multivariable logistic regression regarding associations between *Parabacteroides* abundance and obesity adjusted for age and gender (**A**) and stratified according to sex and age (**B**) in the GGMP cohort. Abbreviations: OR, odds ratio; CI, confidence interval.

In the GGMP cohort, *Parabacteroides* abundance was inversely associated with the prevalence of obesity, with an adjusted OR of 0.43 (95 CI: 0.34–0.56) for Q4 compared with Q1. Subgroup analysis according to sex and age showed a difference regarding the associations between *Parabacteroides* abundance and obesity. Compared with the Q1 of *Parabacteroides* abundance, the ORs (95% CIs) of obesity were 0.71 (0.53–0.95) for Q2, 0.48 (0.35–0.65) for Q3, and 0.34 (0.24–0.47) for Q4 among females, while they were 1.11 (0.78–1.58), 0.81 (0.58–1.18), and 0.62 (0.41–0.93) among males. We observed a significant negative association between *Parabacteroides* abundance and obesity among participants aged 18–39 (Q4 vs. Q1: 0.32, 0.18–0.56) and 40–69 (0.41, 0.30–0.56) but not among those aged 70–90 (1.04, 0.50–2.16) (Figure 3). Four OTUs, including Seq831 (*P. merdae*), *Seq12198* (*P. distasonis*), *Seq9491* (*P. goldsteinii*), and *Seq11608* (*P. gordonii*), were inversely associated with the prevalence of obesity (Supplementary File S1: Figure S3).

**Figure 4:**
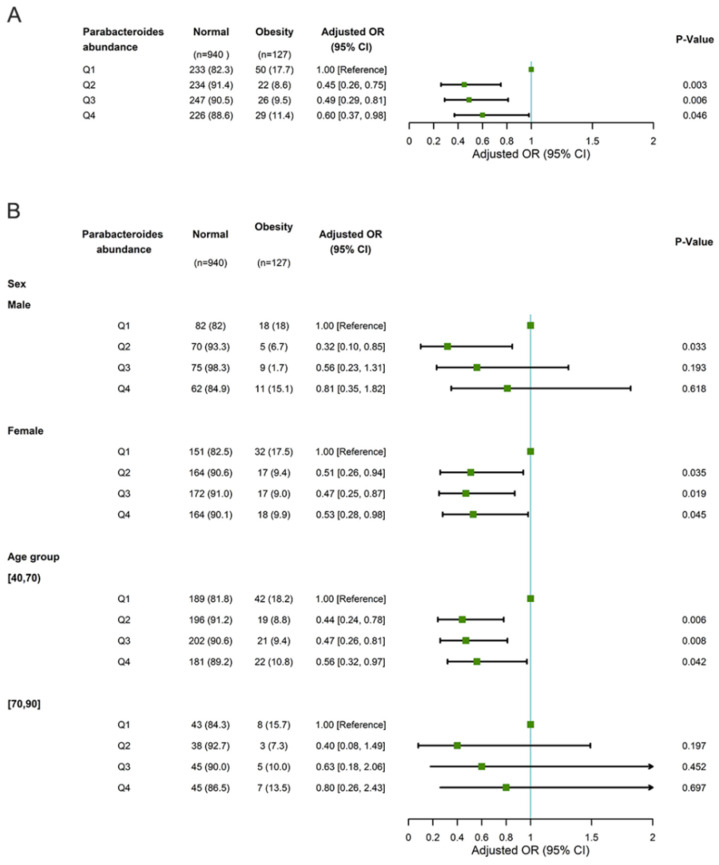
Multivariable logistic regression regarding associations between *Parabacteroides* abundance and obesity adjusted for age and gender (**A**) and stratified according to sex and age (**B**) in the GNSH cohort. Abbreviations: OR, odds ratio; CI, confidence interval.

In the GNHS cohort, *Parabacteroides* abundance was inversely associated with the prevalence of obesity, with an adjusted OR of 0.60 (0.37–0.98) for Q4 compared with Q1. An inverse association between *Parabacteroides* abundance and obesity was found, with an adjusted OR of 0.53 (0.28–0.98) for Q4 among females and 0.56 (0.32–0.97) among participants aged 40–69 years but not among males nor participants aged 70–90 years (Figure 4). However, we failed to identify significant associations between the main *OTUs* and the prevalence of obesity (Supplementary File S1: Figure S4).

### 3.3. The Association of Parabacteroides Abundance with New-Onset Overweight/Obesity and No Longer Being Overweight/Obese

Of the 697 participants with a normal weight at baseline in the GNSH, 85 participants developed overweight/obesity, while the status of 612 participants had not changed at the follow-up. The logistic model showed that there were no significant associations between the abundance of *Parabacteroides* or *OTUs* and the incidence of overweight/obesity compared with the maintaining normal weight group (Table 2). In the same way, of the 546 overweight/obese patients at the baseline, 76 had become normal weight, while 470 participants were still overweight/obese after 3 years of visits. We failed to identify significant associations between the abundance of *Parabacteroides* or *OTUs* and the cessation of overweight/obesity after a 3-year follow-up (Table 3).

### 3.4. Prospective Association between Long-Term Weight Change Pattern and Parabacteroides in GNHS

The linear regression models presented no significant correlations between the abundance of *Parabacteroides* and changes in BMI (*p* > 0.05), regardless of adjusting for age or gender (Table 4). There was no significant correlation between *Seq831* (*P. merdae*), *Seq12198* (*P. distasonis*), *Seq4023* (*P. distasonis*), and changes in BMI in the overall population or across subgroups (*p* > 0.05). Despite the lack of a significant association between *Seq4172* (*P. johnsonii*) and BMI change, the results of the stratified analyses suggested an inverse correlation in the female (Linear regression coefficient = −30.67, *p* = 0.021) and middle-aged populations (Linear regression coefficient = −27.74, *p* = 0.013) but not in the male (Linear regression coefficient = 4.35, *p* = 0.823) or elderly (Linear regression coefficient = −11.17, *p* = 0.694) populations after adjusting for age and gender (Table 4).

## 4. Discussion

In the present large-scale population-based cross-sectional study, we found that the abundance of *Parabacteroides* was inversely associated with obesity, especially in the female and middle-aged populations, and we successfully replicated this association in another cross-sectional dataset. However, the results of our study based on the longitudinal GNSH cohort failed to indicate that higher *Parabacteroides* abundance was associated with a decreased new-onset overweight/obesity risk among normal weight participants, although it was shown to be associated with an increased obesity recovery rate among overweight/obese participants. In the subgroup analysis, *Seq4172* (*P. johnsonii*) indeed showed a negative prospective association with subsequent changes in BMI in the female and middle-aged subpopulations. Overall, although the inverse relationship between obesity and *Parabacteroides* was highly significant in this cross-sectional study, further research is required for its verification or to determine whether such an association is modified by age or gender or is specific to a certain microbial strain.

Age is a potential confounder in the etiological inferences of observational studies that can shape the gut microbiota in terms both composition and diversity and confuse relationships between microbiota and diseases [22,23]. A previous report found that the aging-associated microbiome masked the microbial signatures of colorectal cancer because of the interaction between the cancer microbiome and aging [24]. In the present study, we found that the potential protective effect of *Parabacteroides* decreased with aging, and it was not even been observed among elderly people (>70 years old). In accordance, our latest findings suggest that the relationship between the aging trajectory of microbiota and metabolic diseases is age-dependent [25], suggesting that *Parabacteroides* may have different effects on the risk of obesity in different age groups. It is possible that *Parabacteroides* levels in older individuals (>50 y) are positively correlated with the pathways responsible for lipopolysaccharide (LPS) biosynthesis and the degradation of short-chain fatty acids (SCFAs) [26], which affect immune status and inflammation among older adults.

The gender differences in relation to the gut microbiota are well known. A previous study revealed that the abundance of *Parabacteroides* was only decreased in obese girls in 32 case–control samples matched for normal weight and obesity via 16S rRNA gene sequencing [27]. In contrast, Wang et al. found that the abundance of *Parabacteroides* was higher in adult males in 19 case–control samples analyzed via 16S rRNA gene sequencing [28]. In the present study, we found that the association between *Parabacteroides* and obesity was stronger in females than in males in the GGMP cohort, and the adjusted OR was statistically significant in females but not in males in the GNSH cohort. It is possible that the gut microbiota is modulated by estrogens, especially since the number of *Parabacteroides* significantly increased after the treatment of mice with estrogens; moreover, some metabolic agents, like bile acids, can be hydrolyzed by *Parabacteroides* and converted into secondary bile acids to alleviate lipid metabolism disorders [29,30].

According to the sequence-level analysis of *Parabacteroides*, there are five common OTUs of *Parabacteroides* in the two cohorts, namely, *Seq831* (*P. merdae*), *Seq12198* (*P. distasonis*), *Seq4023* (*P. goldsteinii*), *Seq15333* (*P. gordonii*), and *Seq4172* (*P. johnsonii*). The abundances of their OTUs also accounted for more than 80% of the OTUs in the GGMP and GNHS cohorts. However, there exist inconsistent correlations between OTUs and host health conditions; for example, *Seq12198* and *Seq4023* all belong to *P. distasonis*, but they have different associations with BMI and WC. This discrepancy could be due to the fact that the below-species-level taxa of *Parabacteroides* cannot be detected using the current detection methods (e.g., qPCR sequencing, whole-genome shotgun sequencing, and 16S rRNA sequencing). There is little research on the impact of *P. johnsonii* on obesity. In our study, *Seq4172* (*P. johnsonii*) showed an inverse association with changes in BMI in the GNSH cohort during the 3-year follow-up when adjusting for age, gender, and initial BMI, while the latest study [31], an animal experiment, has shown that *P. johnsonii* can induce the growth of CD8 T cells, which produce interferon-γ and strengthen the anti-tumor immune response. The above studies indicate that the relationship between non-*P. distasonis* and obesity should be scrutinized. *Seq4172* (*P. johnsonii*) should be isolated from the feces of humans and used in obese mice experiments in future studies.

The strengths of the present study are as follows. First, we explored the relationship between *Parabacteroides* and obesity in different subpopulations, e.g., different ages and sexes, based on a large probability proportional sampling cross-sectional study, and the sufficient and representative sample ensured the statistical power of this study. Furthermore, the above findings were validated in another large cohort study. Second, for the first time, we investigated the relationship between *Parabacteroides,* along with its main *OTUs*, and changes in BMI after 3-year follow-ups in a prospective cohort study. However, there were also some limitations of our study. First, the ratio of males to females in the GNSH was female-biased. Second, both the GGMP and GNHS were conducted throughout Guangdong Province in South China, but more participants from other districts should have been recruited to assess the generalizability of the results. Third, the relative abundance of *Parabacteroides* was measured via 16S rRNA sequencing, which will be investigated in the future with quantitative PCR to measure its absolute quantity and its relationship with obesity.

In conclusion, the cross-sectional associations between *Parabacteroids* and obesity are much more pronounced in female and younger individuals than in male and elderlies. Longitudinal studies are needed to investigate the prospective relationship between *Parabacteroides* species and obesity risk, especially in different sex and age subpopulations.

## Figures and Tables

**Table 1:** Basic characteristics of the participants from the GGMP.

Characteristics	Overall	Q1	Q2	Q3	Q4	*p*-Value
No. of participants	5843	1416	1408	1440	1433	
Age, year ^a^	52.98 (14.34)	54.37 (13.74)	53.51 (13.85)	52.54 (14.57)	51.39 (15.04)	<0.001
Male (n, %)	2598 (44.5)	645 (45.6)	638 (45.3)	647 (44.9)	612 (42.7)	0.4
BMI, kg/m^2 a^	23.78 (3.23)	24.22 (3.36)	23.97 (3.33)	23.52 (3.14)	23.41 (3.02)	<0.001
WC, cm ^a^	81.27 (9.36)	82.82 (9.39)	81.52 (9.68)	80.62 (9.21)	80.12 (8.99)	<0.001
SBP, mmHg ^a^	132.24 (21.78)	135.59 (22.13)	133.16 (21.99)	131.24 (21.59)	128.76 (20.91)	<0.001
DBP, mmHg ^a^	77.98 (11.49)	79.34 (11.28)	78.49 (11.79)	77.70 (11.33)	76.47 (11.34)	<0.001
FBG, mmol/L ^b^	5.32 [4.93, 5.83]	5.42 [4.98, 5.93]	5.36 [4.98, 5.84]	5.30 [4.90, 5.81]	5.26 [4.87, 5.72]	<0.001
HbA1c ^b^	4.90 [4.40, 5.30]	4.90 [4.40, 5.30]	4.90 [4.50, 5.30]	4.90 [4.50, 5.30]	4.90 [4.50, 5.30]	0.62
TC, mmol/L ^a^	5.26 (0.85)	5.34 (0.87)	5.29 (0.84)	5.24 (0.87)	5.16 (0.84)	<0.001
Tg, mmol/L ^b^	1.10 [0.76, 1.64]	1.16 [0.80, 1.71]	1.10 [0.76, 1.70]	1.05 [0.75, 1.56]	1.09 [0.74, 1.63]	<0.001
HDL-C, mmol/L ^b^	1.22 [1.00, 1.47]	1.21 [0.98, 1.46]	1.22 [1.00, 1.46]	1.22 [1.01, 1.48]	1.21 [1.00, 1.47]	0.343
LDL-C, mmol/L ^a^	3.28 (0.95)	3.31 (0.97)	3.28 (0.92)	3.29 (0.95)	3.24 (0.95)	0.188
UA, μmol/L ^a^	338.18 (91.98)	345.15 (93.77)	341.33 (93.40)	332.25 (91.69)	333.73 (88.46)	<0.001

^a^ Data are presented as means (SD). ^b^ Data are presented as medians (IQR). Otherwise, the data are presented as numbers (%). BMI, body mass index; WC, waist circumference; SBP, systolic blood pressure; DBP, diastolic blood pressure; FBG, fasting blood glucose; HbA1c, glycated hemoglobin; TC, total cholesterol; Tg, triglyceride; HDL-C, high-density lipoprotein cholesterol; LDL-C, low-density lipoprotein cholesterol; UA, uric acid.

**Table 2:** Associations between *Parabacteroides* and incidence of overweight/obesity after a 3-year follow-up.

Variable	Risk Ratio * (95% CI)	*p* Value
*Parabacteroides*	0.51 (0.23, 1.32)	0.134
*Seq4172_ P. johnsonii*	0.89 (0.36, 1.91)	0.786
*Seq831_ P. merdae*	1.20 (0.76, 1.91)	0.440
*Seq12198_ P. distasonis*	0.91 (0.53, 1.50)	0.716
*Seq4023_ P. distasonis*	1.20 (0.61, 2.21)	0.571
*Seq6030_ P. johnsonii*	0.94 (0.51, 1.62)	0.831
*Seq15333_ P. gordonii*	0.58 (0.24, 1.22)	0.185

* The Logistic regression models were adjusted for age and sex. The lowest quartile (Q1) of abundance was defined as the reference group, and only the OR along with the 95% CIs for Q4 are shown.

**Table 3:** Associations between *Parabacteroides* and the cessation of overweight/obesity after a 3-year follow-up.

Variable	Risk Ratio * (95% CI)	*p* Value
*Parabacteroides*	0.73 (0.17, 2.16)	0.618
*Seq4172_ P. johnsonii*	0.76 (0. 38, 1.66)	0.459
*Seq831_ P. merdae*	0.72 (0.43, 1.18)	0.196
*Seq12198_ P. distasonis*	1.10 (0.64, 1.95)	0.725
*Seq4023_ P. distasonis*	0.96 (0.51, 1.96)	0.911
*Seq6030_ P. johnsonii*	1.46 (0.75, 3.13)	0.289
*Seq15333_ P. gordonii*	0.82 (0.40, 1.88)	0.87

* The Logistic regression models were adjusted for age and sex. The lowest quartile (Q1) of abundance was defined as the reference group, and only the OR along with the 95% CIs for Q4 are shown.

**Table 4:** Multivariable-adjusted associations between the main OTUs abundance of *Parabacteroides* and the change in BMI from the baseline values to the first follow-up (△BMI) in the GNSH cohort.

The Main OTUs of *Parabacteroides*												
Subgroup	*Parabacteroides*		*Seq831*		*Seq12198*		*Seq4023*		*Seq6030*		*Seq4172*	
Overall (*n* = 1243)	β Coefficient (95% CI)	*p* Value	β Coefficient (95% CI)	*p* Value	β Coefficient (95% CI)	*p* Value	β Coefficient (95% CI)	*p* Value	β Coefficient (95% CI)	*p* Value	β Coefficient (95% CI)	*p* Value
Model 1	−2.40 (−6.79, 1.98)	0.283	−0.01 (−5.12, 5.09)	0.996	−2.86 (−14.543, 8.822)	0.631	−5.307 (−18.511, 7.897)	0.431	22.54 (−11.31, 56.39)	0.192	−22.94 (−44.73, −1.14)	0.039
Model 2	−2.46 (−6.84, 1.92)	0.271	−0.27 (−5.45, 4.91)	0.919	−2.083 (−13.896, 9.730)	0.729	−5.176 (−18.450, 8.099)	0.444	22.34 (−11.34, 56.12)	0.195	−24.052 (−43.90, −0.38)	0.046
Female (*n* = 808)												
Model 1	−0.38 (−5.91, 5.14)	0.891	2.77 (−4.01, 9.57)	0.422	−4.75 (−18.89, 9.38)	0.509	−4.35 (−23.50, 14.79)	0.655	23.92 (−12.74, 60.59)	0.201	−31.30 (−57.27, −5.32)	0.018
Model 2	−0.63 (−6.15, 4.88)	0.821	2.61 (−4.16, 9.40)	0.449	−5.54 (−19.66, 8.58)	0.441	−2.73 (−21.88, 16.42)	0.780	22.98 (−13.62, 59.59)	0.218	−30.67 (−56.59, −4.74)	0.021
Male (*n* = 435)												
Model 1	−4.42 (−10.69, 1.84)	0.166	−6.42 (−13.93, 1.09)	0.094	8.81 (−13.95, 31.59)	0.447	−6.97 (−23.44, 9.49)	0.406	45.96 (−5.70, 97.64)	0.081	2.93 (−35.47, −41.35)	0.881
Model 2	−4.20 (−10.45, 2.03)	0.186	−6.17 (−13.64, 1.30)	0.106	11.26 (−11.44, 33.96)	0.330	−5.63 (−22.05, 10.75)	0.50	39.01 (−12.80, 90.81)	0.140	4.35 (−33.86, 42.57)	0.823
40–69 years old (*n* = 1032)												
Model 1	−1.21 (−5.57, 3.13)	0.583	1.25 (−4.02, 6.53)	0.640	−4.90 (−16.97, 7.16)	0.425	−7.15 (−20.83, 6.52)	0.305	19.11 (−9.42, 47.64)	0.189	−28.7 (−51.37, 6.07)	0.013
Model 2	−1.27 (−5.63, 3.07)	0.565	1.18 (−4.09, 6.45)	0.660	−5.02 (−17.08, 7.03)	0.424	−6.24 (−19.94, 7.44)	0.370	17.85 (−10.69, 46.40)	0.22	−27.74 (−50.41, 5.08)	0.016
70–90 years old (*n* = 211)												
Model 1	−2.71 (−15.42, 10.03)	0.676	−5.99 (−21.98, 10.00)	0.461	9.47 (−26.09, 45.04)	0.60	−1.85 (−40.94, 37.24)	0.926	130.44 (−11.96, 272.85)	0.072	−6.94 (−63.87, 49.98)	0.81
Model 2	−3.03 (−15.53, 9.46)	0.539	−4.91 (−20.62, 10.81)	0.539	8.23 (−26.69, 43.14)	0.643	3.59 (−34.93, 42.12)	0.854	122.64 (−18.14, 263.41)	0.087	−11.17 (−67.09, 44.74)	0.694

Model 1: unadjusted regression model. Model 2: adjusted for age and sex.

## Data Availability

The raw data of metagenomic sequencing came from the Guangdong Gut Microbiome Project (GGMP). The raw data for 16SrRNA gene sequences are available from the European Nucleotide Archive (https://www.ebi.ac.uk/ena, accessed on 1 December 2021) under the accession number PRJEB18535. The raw data of metagenomic sequencing from the GNHS are available in the CNSA (https://db.cngb.org/cnsa/, accessed on 31 January 2021) of CNGBdb at accession number CNP0001510.

## Supplementary Materials

The following supporting information can be downloaded at: https://www.mdpi.com/article/10.3390/microorganisms11082087/s1, Figure S1. Distribution characteristics of *Parabacteroides* in GGMP; Figure S2. Distribution characteristics of *Parabacteroides* in GNSH; Figure S3. Multivariable Logistic regression for associations of the main OUTs of *Parabacteroides* abundance with obesity adjusted for age and gender; Figure S4. Multivariable Logistic regression for associations of the main OUTs of *Parabacteroides* abundance with obesity adjusted for age and gender. Table S1. Basic characteristics of the participants from GNSH.
