# Supplementary material for: Sex- and Age-Dependent Associations between Parabacteroides and Obesity: Evidence from Two Population Cohort
Source: Microorganisms. 2023 Aug 15;11(8):2087. doi: 10.3390/microorganisms11082087 (PMC10459623; doi:10.3390/microorganisms11082087)
Supplement: Supplementary file 1 [file microorganisms-11-02087-s001.zip › microorganisms-2425837-supplementary/supplement materila and figure/Supplemetary Materials File.pdf]

Supplementary materials

Table S1. Basic characteristics of the participants from GNSH

Figure S1. Distribution characteristics of Parabacteroides in the population in GGMP

Figure S2. Distribution characteristics of Parabacteroides in the population in GNSH

Figure S3. Multivariable Logistic regression for associations of the main OUTs of Parabacteroides abundance with obesity adjusted for age and gender

Figure S4. Multivariable Logistic regression for associations of the main OUTs of Parabacteroides abundance with obesity adjusted for age and gender

Table s1. Baseline characteristics of the participants in GNSH

Characteristics	Overall	Q1	Q2	Q3	Q4	P-Value
No. of participants	1637	188	294	411	655	
Age, year [a]	63.98 (6.07)	63.76 (5.68)	63.82 (5.95)	63.61 (5.78)	64.15 (6.20)	0.523
Male (n, %)	557 (34.0)	63 (33.5)	100 (34.0)	138 (33.6)	222 (33.9)	0.99
Height, cm [a]	158.40 (7.70)	158.57 (7.83)	158.43 (7.89)	158.45 (7.65)	158.38 (7.72)	0.993
Weight, kg [a]	59.67 (9.33)	60.53 (9.28)	59.94 (9.58)	59.16 (8.89)	59.51 (9.57)	0.362
BMI, kg/m^2 [a]	23.72 (2.91)	24.02 (2.84)	23.86 (3.29)	23.50 (2.64)	23.65 (2.83)	0.152
WC, cm [a]	89.01 (9.68)	91.50 (8.28)	89.65 (12.60)	85.20 (8.89)	88.81 (10.70)	0.768
SBP, mmHg [a]	121.37 (17.03)	121.99 (16.56)	122.67 (17.17)	120.27 (16.83)	121.29 (17.39)	0.301
DBP, mmHg [a]	74.45 (10.07)	74.87 (10.83)	75.16 (10.58)	73.74 (9.59)	74.80 (10.04)	0.236
FBG, mmol/L [b]	5.11 [4.74, 5.57]	5.14 [4.76, 5.76]	5.14 [4.81, 5.56]	5.10 [4.76, 5.58]	5.11[4.72, 5.55]	0.664
HbA1c [b]	5.70 [5.40, 5.90]	5.60 [5.40, 6.00]	5.70 [5.40, 6.00]	5.60 [5.40, 5.90]	5.60 [5.40, 5.90]	0.077
TC, mmol/L [a]	5.60 (1.11)	5.58 (1.29)	5.51 (1.04)	5.61 (1.04)	5.65 (1.09)	0.337
Tg, mmol/L [b]	1.30 [0.96, 1.83]	1.46 [1.02, 2.08]	1.27 [0.98, 1.89]	1.25 [0.93, 1.79]	1.29 [0.96, 1.80]	0.046

Characteristics	Overall	Q1	Q2	Q3	Q4	P-Value
HDL-C, mmol/L [b]	1.43 [1.20, 1.67]	1.42 [1.16, 1.64]	1.41 [1.19, 1.67]	1.45 [1.22, 1.70]	1.45 [1.21, 1.68]	0.172
LDL-C, mmol/L [a]	3.60 (0.96)	3.57 (1.10)	3.52 (0.88)	3.59 (0.91)	3.64 (0.96)	0.336
UA, μmol/L [a]	206.24 [151.00, 286.07]	213.00[157.99, 284.45]	206.50[148.07, 276.60]	201.23[151.10, 293.94]	205.70 [148.13, 286.90]	0.796

[a] Data are presented as the mean (SD).

[b] Data are presented as median [IQR]. Otherwise, the data are presented as number [%]

BMI, body mass index; WC, waist circumference; SBP, systolic blood pressure; DBP, diastolic blood pressure; FBG, fasting blood glucose; TC, total cholesterol; HDL-C, HDL cholesterol; LDL-C, LDL cholesterol; UA, uric acid.

BMI, body mass index; WC, waist circumference; SBP, systolic blood pressure; DBP, diastolic blood pressure; FBG, fasting blood glucose; HbA1c, glycated hemoglobin; TC, total cholesterol; Tg, triglyceride; HDL-C, high-density lipoprotein cholesterol; LDL-C, low-density lipoprotein cholesterol; UA, uric acid.
