# Supplementary figures and images for: Sex- and Age-Dependent Associations between Parabacteroides and Obesity: Evidence from Two Population Cohort
Source: Microorganisms. 2023 Aug 15;11(8):2087. doi: 10.3390/microorganisms11082087 (PMC10459623; doi:10.3390/microorganisms11082087)

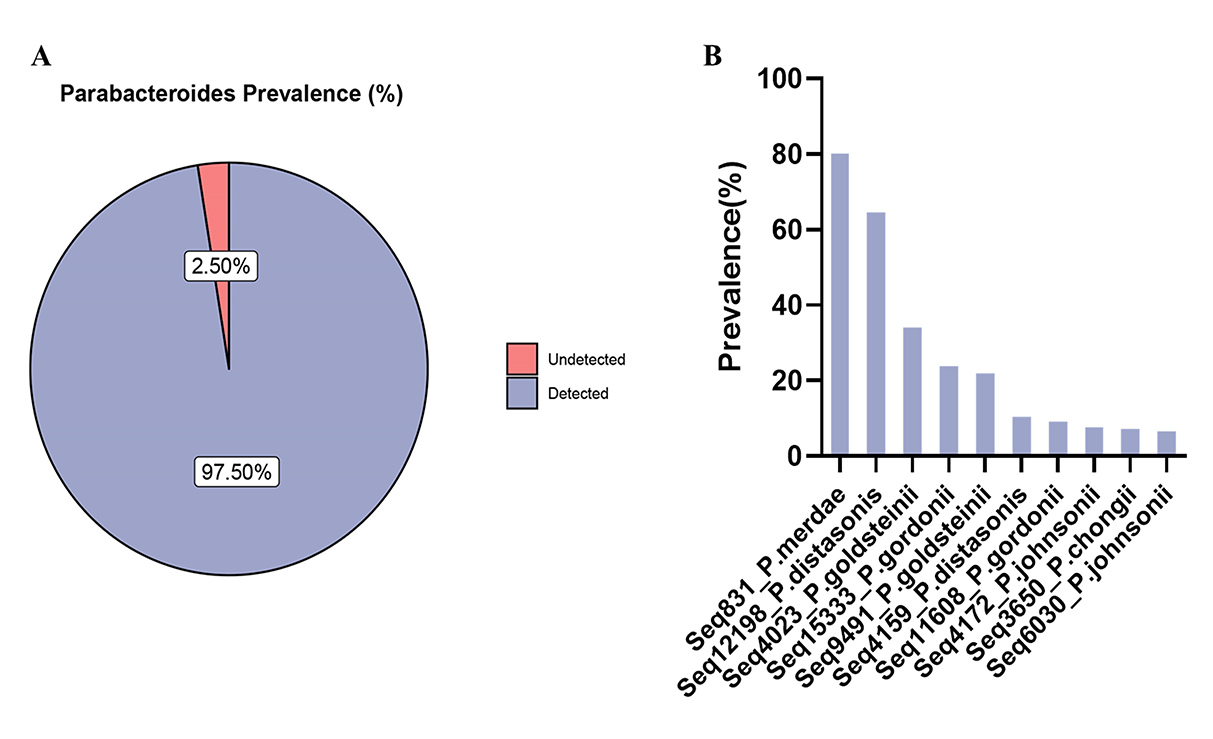

Supplement: Supplementary file 1 [file microorganisms-11-02087-s001.zip › microorganisms-2425837-supplementary/supplement materila and figure/Figure s1.png]

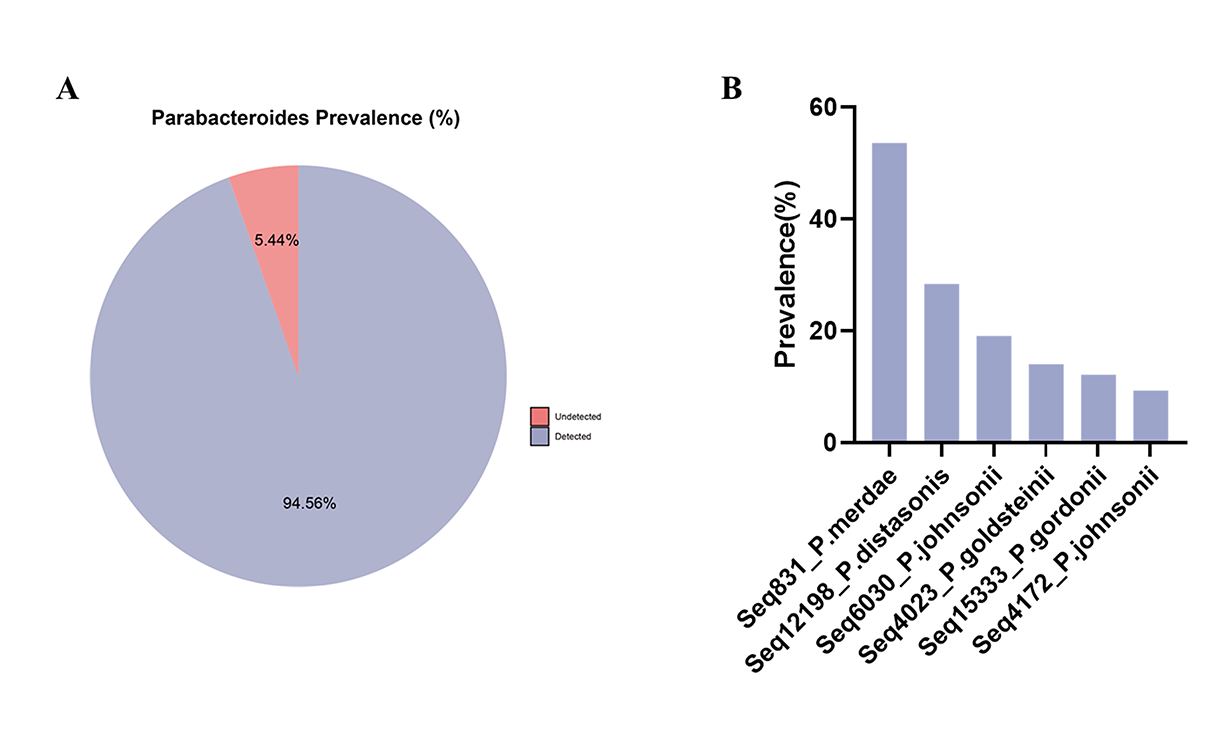

Supplement: Supplementary file 1 [file microorganisms-11-02087-s001.zip › microorganisms-2425837-supplementary/supplement materila and figure/Figure s2 .png]

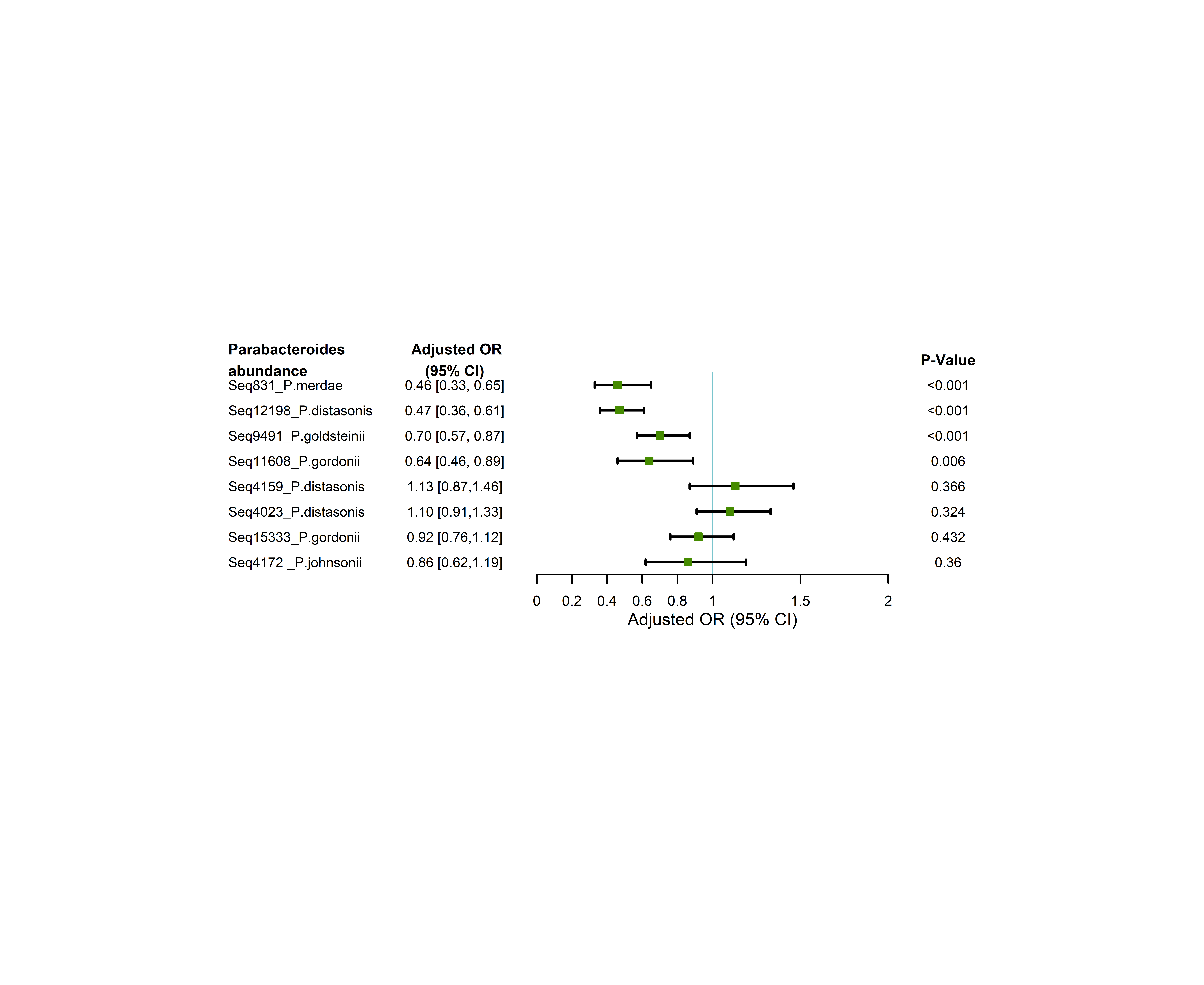

Supplement: Supplementary file 1 [file microorganisms-11-02087-s001.zip › microorganisms-2425837-supplementary/supplement materila and figure/Figure s3.png]

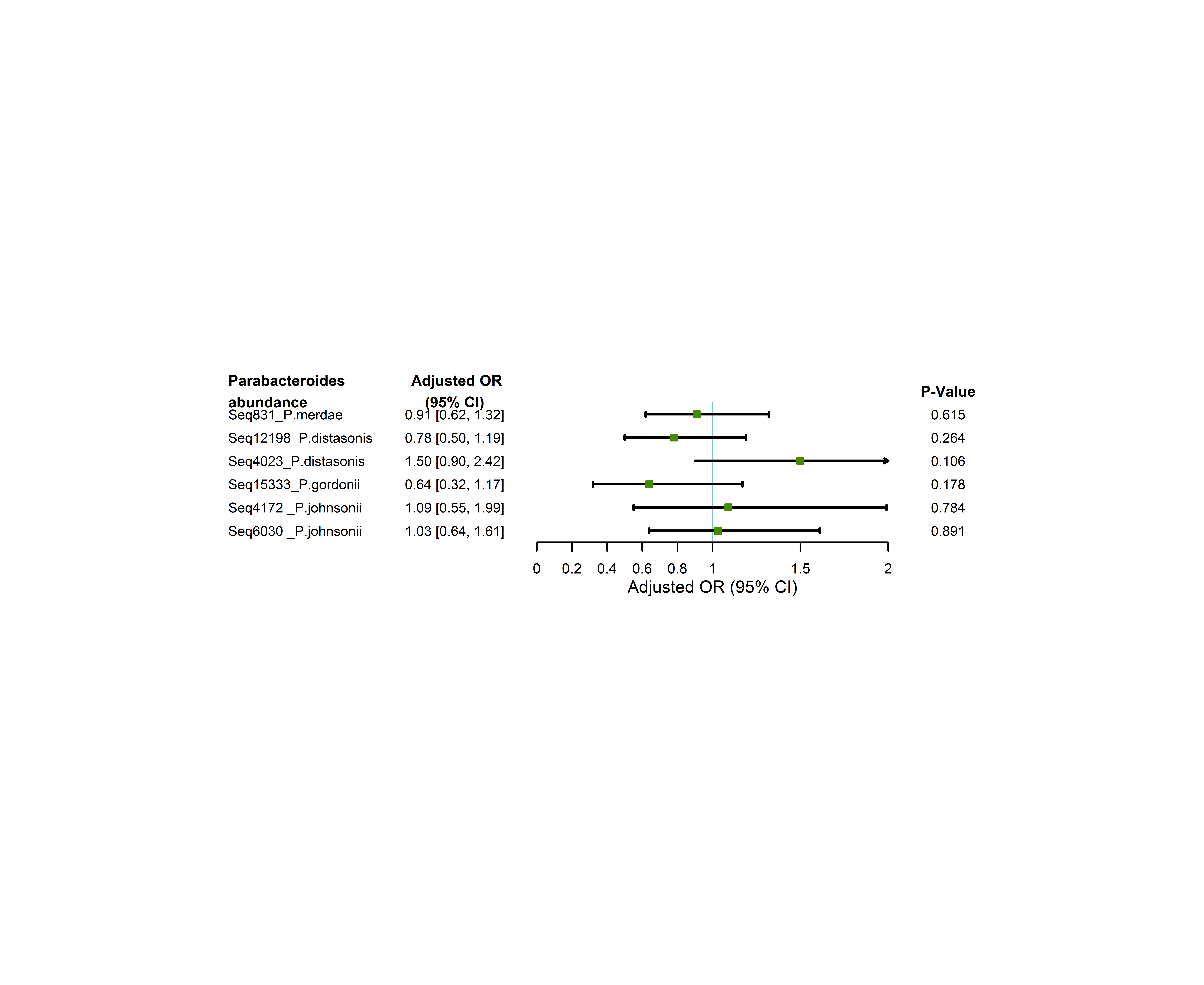

Supplement: Supplementary file 1 [file microorganisms-11-02087-s001.zip › microorganisms-2425837-supplementary/supplement materila and figure/Figure s4.png]
